# Extracting Medical Information From Free-Text and Unstructured Patient-Generated Health Data Using Natural Language Processing Methods: Feasibility Study With Real-world Data

**DOI:** 10.2196/43014

**Published:** 2023-03-07

**Authors:** Emre Sezgin, Syed-Amad Hussain, Steve Rust, Yungui Huang

**Affiliations:** 1 The Abigail Wexner Research Institute at Nationwide Children's Hospital Columbus, OH United States; 2 The Ohio State University College of Medicine Columbus, OH United States

**Keywords:** patient-generated health data, natural language processing, named entity recognition, patient health records, text notes, voice, audio real-world data

## Abstract

**Background:**

Patient-generated health data (PGHD) captured via smart devices or digital health technologies can reflect an individual health journey. PGHD enables tracking and monitoring of personal health conditions, symptoms, and medications out of the clinic, which is crucial for self-care and shared clinical decisions. In addition to self-reported measures and structured PGHD (eg, self-screening, sensor-based biometric data), free-text and unstructured PGHD (eg, patient care note, medical diary) can provide a broader view of a patient’s journey and health condition. Natural language processing (NLP) is used to process and analyze unstructured data to create meaningful summaries and insights, showing promise to improve the utilization of PGHD.

**Objective:**

Our aim is to understand and demonstrate the feasibility of an NLP pipeline to extract medication and symptom information from real-world patient and caregiver data.

**Methods:**

We report a secondary data analysis, using a data set collected from 24 parents of children with special health care needs (CSHCN) who were recruited via a nonrandom sampling approach. Participants used a voice-interactive app for 2 weeks, generating free-text patient notes (audio transcription or text entry). We built an NLP pipeline using a zero-shot approach (adaptive to low-resource settings). We used named entity recognition (NER) and medical ontologies (RXNorm and SNOMED CT [Systematized Nomenclature of Medicine Clinical Terms]) to identify medication and symptoms. Sentence-level dependency parse trees and part-of-speech tags were used to extract additional entity information using the syntactic properties of a note. We assessed the data; evaluated the pipeline with the patient notes; and reported the precision, recall, and *F*_1_ scores.

**Results:**

In total, 87 patient notes are included (audio transcriptions n=78 and text entries n=9) from 24 parents who have at least one CSHCN. The participants were between the ages of 26 and 59 years. The majority were White (n=22, 92%), had more than one child (n=16, 67%), lived in Ohio (n=22, 92%), had mid- or upper-mid household income (n=15, 62.5%), and had higher level education (n=24, 58%). Out of 87 notes, 30 were drug and medication related, and 46 were symptom related. We captured medication instances (medication, unit, quantity, and date) and symptoms satisfactorily (precision >0.65, recall >0.77, *F*_1_>0.72). These results indicate the potential when using NER and dependency parsing through an NLP pipeline on information extraction from unstructured PGHD.

**Conclusions:**

The proposed NLP pipeline was found to be feasible for use with real-world unstructured PGHD to accomplish medication and symptom extraction. Unstructured PGHD can be leveraged to inform clinical decision-making, remote monitoring, and self-care including medical adherence and chronic disease management. With customizable information extraction methods using NER and medical ontologies, NLP models can feasibly extract a broad range of clinical information from unstructured PGHD in low-resource settings (eg, a limited number of patient notes or training data).

## Introduction

Patient-generated health data (PGHD) volume is growing immensely with the increased use of digital devices. The Office of the National Coordinator for Health Information Technology defines PGHD as “health-related data created, recorded, or gathered by or from patients or family members or other caregivers to help address a health concern” [1]. Today, PGHD can be collected out of the clinic using medical and consumer-grade mobile devices as passive or active data, such as blood glucose monitors, wearables (heart rate and SPO), and smartphones (physical activity scores and patient-reported data, such as screening survey responses) [2,3]. PGHD is becoming a necessary component of personal health records as well as remote monitoring and is influencing self-care and clinical decisions [4]. Medical systems have the infrastructure available to integrate digital tools generating PGHD with electronic health record systems to enhance the clinical decision process and eventually improve patients’ quality of life and produce better health outcomes [3,5].

In a patient’s journey (especially patients with chronic conditions or special health care needs), physical medical diaries and patient notes have been the primary source of free-text patient information (“unstructured PGHD”), facilitating the collection of health information. With the adoption of smart devices, there is an increased use of personal devices for digital medical diaries and note-taking [6]. In addition, automatic speech recognition, conversational agents, and voice-interactive technologies ease the process of note-taking via natural conversations [7-9]. However, the patient experience, health events, medications, and symptoms captured in personal notebooks or devices are expected to be communicated verbally or written periodically, such as, during clinical visits. Given the limited time and ability to read and communicate patient notes, this information could be underused and create an additional burden [10]. Integrating PGHD into electronic health record systems is an acknowledged contribution, as it can create a more comprehensive view of health conditions and eventually inform shared decision-making [11]. Yet, free-text patient notes or unstructured PGHD integration requires further considerations on clinical workflow and clinical burden [3].

Therefore, a pipeline for processing unstructured PGHD to inform self-care and clinical decision processes is needed and preferable [12]. In the literature, there are a number of studies reporting natural language processing (NLP) applications on clinical notes to identify symptoms and conditions [13,14]. A subset is focusing on electronic patient-authored texts, which are the patient-reported symptoms and conditions that are shared on the web but mostly out of medical records. The studies report that the NLP applications accompanying large public data sets of electronic patient-authored texts (eg, web-based forums or social media posts) are based on rules, machine learning, or a hybrid combination [15]. Rule-based methods are preferable with the use of clinical domain knowledge (eg, ontologies) for increasing accuracy in entity extraction at the expense of generalizability [14]. Machine learning solutions have been effective in extracting word or sentence meaning by using probabilistic models and being structure-agnostic with variations in spelling and grammar [16]. Yet, machine learning models could be resource intensive. Hybrid models leverage the strength of both approaches in terms of combinatorial patterns among words and semantic relationships [17,18] and are adaptive to low-resource settings.

In this paper, we evaluate a hybrid (machine learning + rule-based) NLP pipeline [19] with low-resource unstructured PHGD (ie, not depending on a large data set for training) and report its feasibility. We focus on extracting medication and symptom information, which must be tracked and communicated to patients with chronic conditions. We complete an empirical evaluation of automatic component extraction where we measure the model’s ability to conduct automatic entity extraction using ontologies (medication dose: RxNORM, symptoms: SNOMED CT [Systematized Nomenclature of Medicine Clinical Terms]) [14,20] from the patient note data set. Namely, this NLP pipeline constitutes a rule-based system that leverages the dependency parsing, named entity recognition (NER), and ontology-linking capabilities of pretrained machine learning models (specifically, deep learning and pretrained language models [PLMs]), allowing for increased interpretability, ease of deployment, and generalization.

## Methods

### Overview

Our study reports a secondary data analysis using the data set collected on a prior research project [21].

### Recruitment and Study Setting

A convenience (nonrandom) sampling method was used to invite participants (parents and caregivers) to participate in the study within the network of the complex care clinic at a large pediatric hospital in the midwest. We sent email invitations and announced the research participation opportunity over social media and digital boards at the hospital. The recruitment occurred between October-December 2020. Inclusion criteria for the study were being a parent of a child with one or more complex medical conditions and having an iPhone with iOS 13 or above (or an iPhone 8 or above) during the study period. A total of 41 participants met these criteria and consented to participate. Of these, 24 participants completed the full study, which included a 2-week period of app use.

### Data Collection

**Figure 1:**
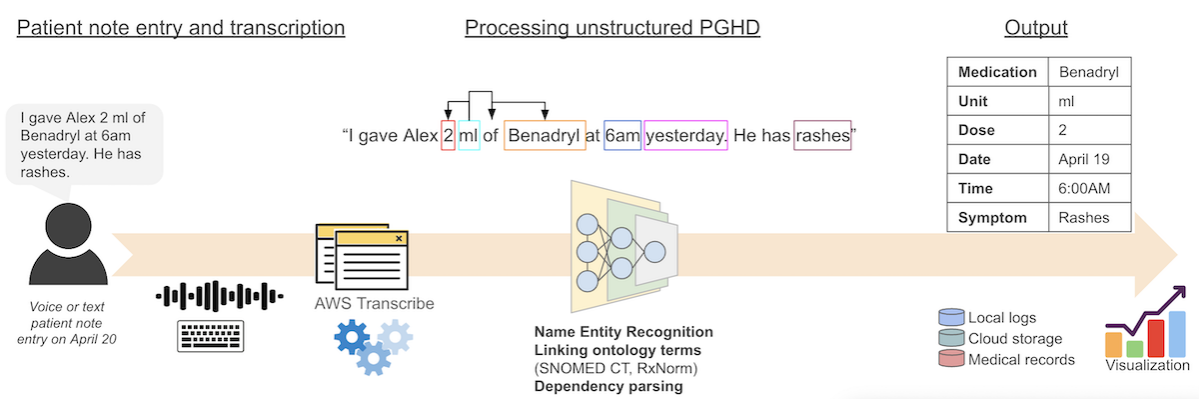
Data collection, processing, and output flow. AWS: Amazon Web Services; PGHD: patient-generated health data; SNOMED CT: Systematized Nomenclature of Medicine Clinical Terms.

Data were collected from 24 parents of children with special health care needs (CSHCN) between October 2020 and January 2021. Participants were onboarded to the study via a web-based screening and survey tool. The eligible participants were guided through a web-based tutorial to install and use the research app. The app has functions to record, transcribe, and store notes [21]. Participants were instructed to use the app for a 2-week period for medical note-taking. During the study period, participants received periodic (every 2-3 days) reminders and tips about how to use the app features. They had the option to create medical note entries through voice or text while at home. Voice entries were transcribed by using Amazon Web Services Transcribe services [22], and the transcriptions were used as patient notes for analysis (Figure 1).

### Data Analysis

Building upon an earlier NLP pipeline proposal with simulated notes [19], we created a pipeline that leverages NER to identify and map terms to existing ontologies, particularly the RXNorm and SNOMED CT ontologies for medication and symptoms, respectively. After that, our algorithm searches over the sentence-level dependency parse trees alongside part-of-speech tags to extract further entity information based on the syntactic properties of the note [19]. Additionally, the relative date of the note (the date reported in the notes), if different from the authorship date, is derived with the assistance of the dateparser python library [23]. The NER and dependency parsing tools are retrieved from the open-source SciSpaCy Python package, which consists of PLM and ML models [24]. More specifically, our pipeline uses the SpaCy en_core_sci_lg model, which is a pipeline for biomedical data leveraging word embedding with more than 780,000 vocabulary terms and 600,000 word vectors [24], and a set of tools that use deep learning models to process biomedical and clinical text [25,26]. Researchers (SAH, ES) compared the model output against the original sentence and assessed it. They marked the information that the model incorrectly extracted (false positive), incorrectly left behind (false negative), and correctly extracted (true positive and true negative) for the categories of medication instance and symptom. We then calculated precision, recall, and combined *F*_1_ score. This pipeline uniquely seeks to extract information about medication and symptoms using a zero-shot approach, which requires no training data and is adaptive to low-resource settings [27]. Likewise, our study does not have standardized tasks; therefore, no other baseline pipelines are available to compare against it. For this reason, evaluation metrics are presented solitarily to enable the evaluation and feasibility of the approach.

Figure 1 illustrates the data collection, processing, and output flow. In summary, a patient or caregiver creates the notes through voice interaction or text entries. Amazon Web Services transcribes voice entries and stores. The entries, unstructured PGHD, are processed using the proposed NLP model in this study to identify medication amount, dose, medication name (dependency parsing is shown with arrows), time and date, and symptoms. The extracted information can be integrated into a chart that can be used to inform a patient, caregiver, and provider through local logs on a device, cloud services, or medical records assisted by data visualization tools [19]. The integration component is not in the scope of this study.

### Ethics Approval

The study involves human participants and was reviewed and approved by the institutional review board at Nationwide Children’s Hospital (#00000231). The participants signed written informed consent to participate in this study, allowing the use of the data set for the data analyses described here. All data reported in this study are deidentified. Participants were compensated for their time with a gift card (US $30).

## Results

### Participant Demographics

The participants were aged between 26 and 59 years (mean 39, median 38), mostly White (n=22, 92%), had more than one child (n=16, 67%), lived in Ohio (n=22, 92%), had mid or upper-mid household income (n=15, 62.5%), and received higher-level education (n=24, 58%). Participants had CSHCN with multiple chronic conditions. Frequently reported conditions included developmental delay; speech, vision, and physical problems; and genetic and neurological disorders. The majority of parents were “always” or “often” tracking their child’s symptoms, medications, or conditions (n=17, 71%) using an app (n=13, 54%) or patient portal (n=16, 67%).

### Notes Characteristics

In total, 87 patient notes were included (voice entry transcriptions n=78 and text entries n=9). Thirty of the notes are drug and medication-related, and 46 of the notes are symptom-related, but there are overlapping notes having both or none of the symptom and medication information. More specifically, 24 notes have no symptom or medication information; 33 notes have only symptom information but no medication information; 17 notes have only medication information and no symptom information; 13 notes have both medication and symptom information. Each note is structured as 1 to 3 sentences, briefly recording the state of the patient, medication given, and symptoms (see Textbox 1, [21]). Content-wise, a patient note entry may have multiple components (symptom and medication detail), such as a summary of the day instead of multiple notes created throughout a day. Please see Multimedia Appendix 1 for the medication and symptoms captured through the pipeline and their frequencies.

**Sample patient notes [21].**

**Symptoms or health condition**

- “Spot on lip is gone. Overall doing well. Has a runny nose it no fever or any other symptoms.”
- “[patient name] oxygen was still hanging out around 80 today...blood sugar is 127.”

**Medication with dose, unit, and time**

- “Gave [patient name] 2 Benadryl at 6:00 am…[patient name] does not take his medicine after lunch.”
- “Yesterday we started…gabapentin at a rate of 2.6 ml that will continue for one week, then we will switch rate to 2 ml over the course of another week, then 1.6 ml for another week with final rate at 0.6 ml...”

### Evaluation Results

Table 1 presents precision, recall, and *F*_1_ scores. In the table, notes refer to the number of individual notes considered for each data component (eg, medication or symptom-related term). Instances refer to the number of times a data component is considered with allowance for multiple instances per note. A medication instance refers to all subcomponents (medication name, unit of measurement, quantity, and date information) of the medication when present. An instance is correct if all subcomponents are correctly extracted when present and is wrong if any one of the presented subcomponents is wrong. Precision-recall scores of medication information subcomponents provide a granular breakdown per extracted instance. Some of the extracted symptoms could be ambiguous to be classified as a symptom, such as emotional states (eg, “happiness”). We categorized them as symptoms in our research, as mood can affect health conditions.

**Table 1:** Evaluation of extracting medication and symptom information through precision, recall, and *F*_1_ scores.

Data component		Precision	Recall	*F*_1_	Notes, n	Instances, n
**Medication instance**		0.83	0.77	0.80	30	62
	Medication	0.97	0.84	0.90	30	62
	Unit	0.86	0.53	0.66	14	27
	Quantity	0.50	0.19	0.27	9	16
	Date	0.93	0.76	0.84	13	33
Symptom		0.65	0.82	0.72	46	71

At an instance level, which is considered correct if all relevant subcomponents are correctly extracted, the medication-instance extraction pipeline has moderately good performance, with a precision and recall both near 0.8. At the subcomponent level, medication name recognition has the highest performance with high precision, while unit and quantity extraction have low recalls. In medication-related false-positive cases, patient names, or common terms (eg, water) overlap with medication names or ingredients (eg, water irrigation solution) within the RXNorm ontology. Unit and quantity extraction errors are often caused by irregular sentence structures or information split between multiple sentences. Date extraction performs well in both precision and recall (except in cases with phrasal referents, such as “A few days ago”). Both brand names and generic names are identified with errors caused by overlap between patient names and RXNORM. For symptoms, we find recall scores higher than precision, implying that often entities were extracted when they were not proper symptoms (eg, “sitting” and “sign”).

## Discussion

### Principal Findings

We present findings of our proposed NLP pipeline with real-world PGHD to demonstrate the feasibility of its implementation. The results demonstrate that the NLP pipeline performance matches contemporary works with a zero-shot approach for information extraction [28], specifically for most of our targeted information categories of medications and symptoms (with an *F*_1_ score above 0.6). These results cannot be compared directly since the other studies in the literature do not focus on PGHD or the same combination of medication, dosage, and symptom extraction. However, the results indicate acceptable performance when using NER and dependency parsing through open-source and hybrid NLP models. The performance of the pipeline may increase over time with improvements in automatic speech recognition and text prediction and suggestion methods (methods that also use NLP models that are not covered within the scope of this study) [29-31]. However, in this study, the pipeline performance was potentially affected by the transcription errors or typing errors existing in the data set (n=16, 18% of 87 notes had at least one error; errors have not been corrected to contain real-world data features).

This study extends the existing literature [15], presenting the capability of current models to extract key information from patient notes. This approach can inform patients and caregivers out of the clinic toward enabling self-care (eg, improving medical adherence, symptom tracking) and remote monitoring (eg, detection, intervention, and communication) [11]. In clinical practice, the use of such artificial intelligence and machine learning approaches potentially facilitates the inclusion of personal health records into medical records, which can allow the identification of health condition changes and build early detection mechanisms [3,32]. Integrating unstructured PGHD via an NLP pipeline within electronic medical records can also contribute to improving patient-reported outcomes and shared decision-making at the clinic, allowing health care providers to remotely observe health conditions and intervene in a timely manner [33,34].

### Extending Digital Health Technologies for Special Health Care Needs

Considering the patient population with special health care needs and their caregivers that receive care from multiple providers and clinics, there is a continuous need for documentation, medication, and symptom tracking during home care. Timely communication of patient conditions with multiple health care providers is needed but creates additional burden and stress for patients and caregivers given their daily life and workload [35,36]. The literature shows that currently available digital health technologies including mobile apps, SMS text messages, and web portals have been used for patient care management [37-41] and communication of patient medical conditions remotely [41-44]. As digital health technology eases the process of documentation and the tracking of symptoms and medications, NLP approaches can improve the process by enabling the use of natural and preferred language. Furthermore, an NLP pipeline integrated with preferred technologies (text-based and voice-interactive apps, patient portals) can reduce the burden and complexity of accessing personal notes, summarizing and searching patient notes, and reducing the need to learn a technology to complete tasks or take notes, and reduce required attention on a device or modality and time spent on documentation [12,21,45]. In addition, the use of the zero-shot approach demonstrates the ability to use artificial intelligence and machine learning in data-scarce environments (eg, data on rare diseases, data from rural hospitals), which increases the equitable and accessible use of artificial intelligence and machine learning in health care.

### Implementing NLP Pipeline

Our study presents the feasibility of PLM use within zero-shot biomedical settings. Whereas other works require specialized pretraining of NLP models [28,46] or are limited to handling the formalized writing style of the biomedical literature, our approach makes use of more task-general biomedical PLMs to better generalize over the various syntactic forms found in PGHD. Namely, we gather our PLMs from the SciSpacy model suite, which shows high performance in its various capabilities when evaluated on PubMed and clinical notes [25]. We use SciSpacy’s entity extraction capabilities before linking to various ontologies. It is followed by the extraction of additional information related to these entities, such as medication dosage information by leveraging sentence-level dependency parse trees, providing insight into the capabilities and using automatic dependency parsing. This is a novel approach to our study, which has not previously been implemented in biomedical research [47-49]. Furthermore, the performance of our pipeline can be improved through the use of cohort- or patient-specific vocabularies to augment the NER subcomponent, allowing for a human-in-the-loop component to our hybrid model where domain experts can define model parameters and integrate human knowledge [50]. Human-in-the-loop methods span a variety of directions with the general consensus being that such methods allow for compensation of model weaknesses with domain expertise and vice versa, alongside a high ratio of model performance against model creation cost, causing it to be an increasingly important component of applied machine learning [18,50]. Using publicly available models and ontologies improve the dissemination of the model as well as access and customization specific to conditions and patient populations. Since our NLP models are pretrained on clinical data and use publicly available ontologies, replication and scalability of a pipeline have low costs in terms of the requirement for training data, computational power requirements, and expertise.

### Limitations

Our study has several limitations. We did not use fine-tuned models or custom vocabularies, which might improve the performance (eg, missing condition-specific treatments and therapies). In addition, we do not implement and evaluate negation. We are unable to compare the feasibility of our pipeline against other NLP models. This is, in part, due to the lack of comparable and available pipelines and tasks to those presented in this study. However, future work is suggested to compare our pipeline against other PLMs as well as performance comparison against a model fine-tuned on general PGHD and clinical notes. These additional approaches may inform how our pipeline can be improved in the future to improve task completion or generalizability.

Our evaluation process was toward feasibility, rather than only performance assessment. In that regard, we used a nonstandard evaluation process as we annotated the examples coming after we ran the pipeline over these examples. As it was necessary to capture the false positives that the pipeline extracted, certain extracted values were considered partially valid only after the model was seen to have captured them, such as moods being considered symptoms. In these cases, the capture of such entities was not considered a false positive, while omittance was not necessarily considered a false negative. Additionally, for medication and dosage entities, it was unlikely for bias to be introduced due to the definite nature of these values (eg, if “Tylenol” is referenced, it is clearly a medication that should be captured).

Due to the limited text-based entries against voice entries, we were not able to measure the discrepancy of the model performance of extracting entities from written notes versus voice transcriptions.

### Technical Contribution

The first key contribution is the use of pretrained language and deep learning models and the extraction of information using syntax parsing and entity-to-ontology linking in the proposed methodology. To our knowledge, these individual components have yet to be combined into a clinical information extraction pipeline. Second, we focus on PGHD for chronic conditions which has been understudied, and, to our knowledge, no examples exist in the literature regarding the use of a text-processing pipeline with the real-world PGHD from this specific population (CSHCN). While previous studies explore similar NLP pipelines with NER, only a few studies use the automatic dependency parser for further relation and entity extraction [47], layer multiple rounds of dependency parsing to extract general relationships from within scientific literature [49], and use dependency parsing to identify SNOMED CT expressions form clinical notes and [48]. These studies show the promise of using syntactic dependency trees to improve generalizability but do not fit the unstructured format of PGHD and are not focused on extracting values related to chronic care, such as medications and dosages.

To accomplish the task of extracting key care-related information from PGHD (medications and symptoms), we created a pipeline that applies dependency parsing to key terms and relationships for chronic care management. Instead of expanding generalizability through training of a deep learning model, which can often be costly in time and labor, the pipeline can be improved and expanded with a system to allow for user input to inform NER and dependency-based relation extraction systems. This inclusivity of users (eg, clinician, patient, or caregiver) allows for flexibility of modification of our system depending on patient condition or clinical needs [19]. Furthermore, by using publicly available ontologies and models, the proposed pipeline can be replicated, customized, and improved for different cohorts with chronic conditions.

### Future Work

Future work will focus on building and fine-tuning condition-specific models, ontologies, and vocabularies, prototyping PGHD integration to clinical workflow, and improving clinical decision support mechanisms through PGHD-informed visuals.

In addition, we plan to analyze voice and audio characteristics and extract-related features (such as pause rates, pitch, loudness, acoustic and spectral features, and multiple speakers, such as parent and child) [51,52]. Voice analytics will add a new dimension to PGHD analytics by investigating vocal and environmental audio features (markers) with patient notes and building a multimodal pipeline, such as improving transcription quality, improving sentiment analysis, identifying the environmental factors [53,54], and guiding future data collection protocols. Textbox 2 provides a glimpse at the data for the proposed future work with voice analytics.

**Augmenting patient-generated health data with voice analytics pipeline in addition to natural language processing pipeline.**

**Figure 2:**
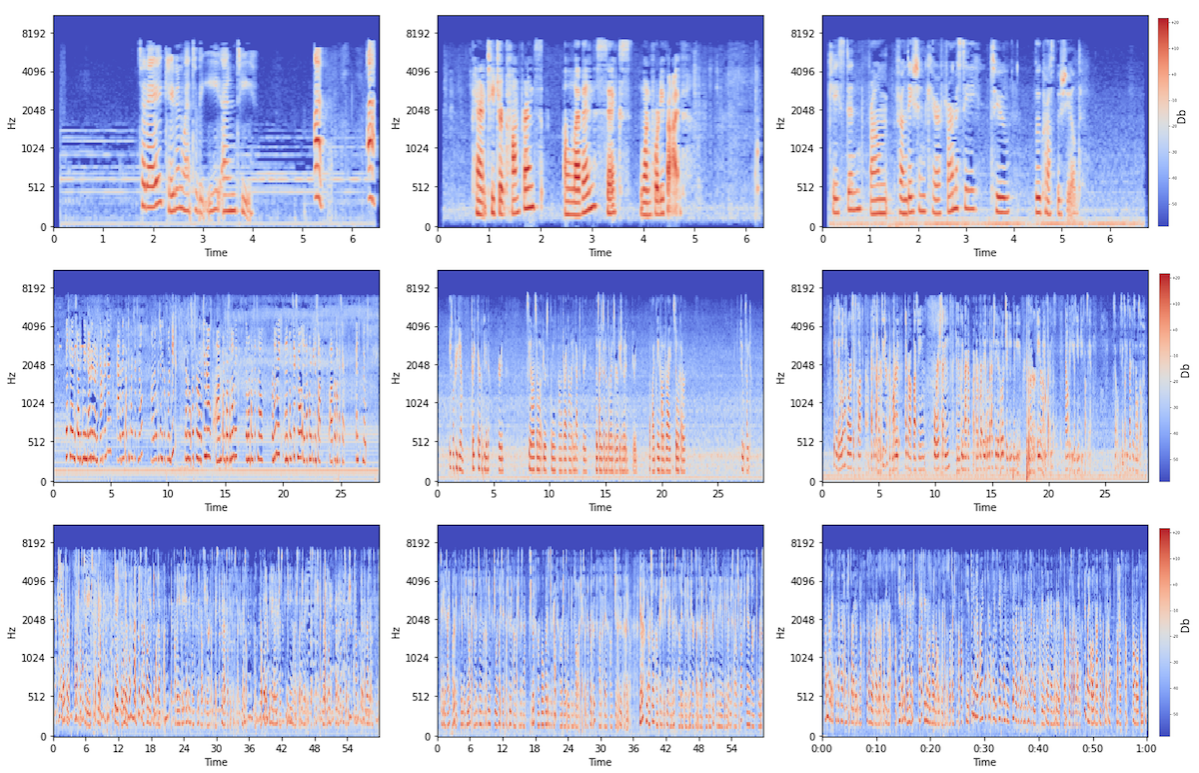
Melspectrogram of participants’ voices. Librosa library was used via Anaconda Spyder (Python 3.10; n_fft=2048, hop_length=512, n_mels=128; audio bitrate at 24 kbps and 20 Hz to 20 kHz frequency range). The top row is from 3 different participants with 6 seconds of recording, the middle row is from 3 different participants with 28 seconds of recording, and the bottom row is from 3 different participants with 60 seconds of recording. The first, third, and sixth melspectograms have higher noise or children sound in the background.

Our study reveals that the voice data have been created in different environments (eg, alone in a silent room, while driving, or in a room with children). This affects the quality of transcriptions and, hence, NLP performance. We plan to transform and analyze audio or voice data with melspectrograms that can help decompose complex features or magnitude of signals of the voice and help extract features efficiently [51]. Eventually, we plan to use convolutional neural network models to extract features and classify them [53]. Figure 2 provides melspectrograms of nine participants’ voice recordings.

### Conclusion

We present the feasibility of an NLP pipeline with real-world data in a low-resource setting, focusing on medication and symptom information extraction. Unstructured PGHD can inform decision-making and support remote monitoring and self-care. With customizable information extraction methods using NER and medical ontologies, NLP models can feasibly extract a broad range of clinical information from unstructured PGHD in low-resource settings. We suggest future work to build medical condition-specific models, test the integration to clinical workflow, and use audio features in the analysis.

## Appendix

Multimedia Appendix 1 Medication and symptom names captured.

## Abbreviations

CSHCN: children with special health care needs
NER: named entity recognition
NLP: natural language processing
PGHD: patient-generated health data
PLM: pretrained language model
SNOMED CT: Systematized Nomenclature of Medicine Clinical Terms

## Data Availability

Because the data sets analyzed during the current study contain personally identifiable information and sensitive health information, they are not publicly available. Research consent did not include making the data available publicly.
