# Supplementary material for: Extracting Medical Information From Free-Text and Unstructured Patient-Generated Health Data Using Natural Language Processing Methods: Feasibility Study With Real-world Data
Source: JMIR Form Res. 2023 Mar 7;7:e43014. doi: 10.2196/43014 (PMC10031450; doi:10.2196/43014)
Supplement: Multimedia Appendix 1 [file formative_v7i1e43014_app1.docx]

Supplementary material: Medication and symptom names captured

| Medication Name | Frequency (occurrence) | Correctly Captured? |  | Symptom Name | Frequency (occurrence) | Correctly Captured? |
| --- | --- | --- | --- | --- | --- | --- |
| Tylenol | 7 | Yes |  | seizure | 7 | Yes |
| medicine | 4 | Yes |  | happy | 5 | Yes |
| gabapentin | 3 | Yes |  | sitting | 3 | No |
| oxycodone | 3 | Yes |  | symptoms | 3 | Yes |
| Ativan | 3 | Yes |  | fever | 3 | Yes |
| desmopressin | 2 | Yes |  | lip | 2 | Yes |
| Bosentan | 2 | Yes |  | headache | 2 | Yes |
| Enalapril | 2 | Yes |  | pain | 2 | Yes |
| pill | 2 | Yes |  | BM | 2 | Yes |
| injection | 2 | Yes |  | agitated | 2 | Yes |
| oxygen | 2 | Yes |  | crying | 2 | Yes |
| Coumadin | 2 | Yes |  | fever blister | 1 | Yes |
| mylicon | 1 | Yes |  | Runny nose | 1 | Yes |
| tylenol | 1 | Yes |  | sweating | 1 | Yes |
| [Patient name] | 1 | No |  | runny nose | 1 | Yes |
| albuterol | 1 | Yes |  | redness | 1 | Yes |
| pills | 1 | Yes |  | sign | 1 | No |
| medication | 1 | Yes |  | No distress | 1 | Yes |
| Humira | 1 | Yes |  | shaking | 1 | Yes |
| water | 1 | No |  | mild | 1 | Yes |
| [Patient name] | 1 | No |  | swollen | 1 | Yes |
| Aspirin | 1 | Yes |  | burning | 1 | Yes |
| Budesonide | 1 | Yes |  | loud | 1 | Yes |
|  |  |  |  | no sign | 1 | No |
|  |  |  |  | catch | 1 | No |
|  |  |  |  | limp | 1 | Yes |
|  |  |  |  | problem | 1 | No |
|  |  |  |  | Right shoulder pain | 1 | Yes |
|  |  |  |  | vomiting | 1 | Yes |
|  |  |  |  | throwing | 1 | Yes |
|  |  |  |  | normal | 1 | Yes |
|  |  |  |  | high blood sugars | 1 | Yes |
|  |  |  |  | gain weight | 1 | Yes |
|  |  |  |  | weak | 1 | Yes |
|  |  |  |  | back pain | 1 | Yes |
|  |  |  |  | night terrors | 1 | Yes |
|  |  |  |  | bleeding | 1 | Yes |
|  |  |  |  | sleepy | 1 | Yes |
|  |  |  |  | painful | 1 | Yes |
|  |  |  |  | wheeziness | 1 | Yes |
